# Embryonic development of parakeratinized epithelium of the tongue in the domestic duck (*Anas platyrhynchos f. domestica*): LM, SEM, and TEM observations

**DOI:** 10.1007/s00709-018-1324-z

**Published:** 2018-10-31

**Authors:** Kinga Skieresz-Szewczyk, Hanna Jackowiak, Marlena Ratajczak

**Affiliations:** 1 Department of Histology and Embryology, Poznań University of Life Sciences, Wojska Polskiego 71C, 60-625 Poznań, Poland; 2 Faculty Laboratory of Electron and Confocal Microscopy, The Adam Mickiewicz University of Poznań, Umultowska 89, 61-614 Poznań, Poland

**Keywords:** Embryonic development, Parakeratinized epithelium, Tongue, Birds, Periderm granules

## Abstract

The parakeratinized epithelium is a common and widespread type of keratinized epithelium in the oral cavity in adult birds. In contrast to orthokeratinized epithelium, which mostly covers mechanical papillae and the lingual nail, parakeratinized epithelium covers almost the entire dorsal surface of the tongue in birds. The characteristic feature of parakeratinized epithelium is the presence of nuclei in the keratinized layer. The present study aimed to investigate for the first time the micro- and ultrastructural changes of parakeratinized epithelium during embryonic development and to assess the readiness of the epithelium to serve protective functions during food transport to the esophagus. Three developmental stages were distinguished: embryonic, transformation, and pre-hatching stages. The embryonic stage lasts from the 9th to the 14th day of incubation and the epithelium is composed of undifferentiated epithelial cells. The transformation stage lasts from the 15th to the 22nd day of incubation and the epithelium undergoes transformation into stratified epithelium consisting of basal, intermediate, and superficial layers. The characteristic feature of this stage is formation of the periderm with osmophilic granules. The pre-hatching stage starts on the 23rd day, and the epithelium with a fully developed keratinized layer resembles that of the epithelium in adult animals. No periderm was observed on the epithelial surface. It was confirmed that at the time of hatching the parakeratinized epithelium is fully differentiated and ready to fulfill its function during food transport. The presence of periderm is a common feature characteristic for para- and orthokeratinized epithelium in the oral cavity of birds. However, the formation of the keratinized/cornified layer is different for these two types of keratinized epithelia.

## Introduction

The lingual mucosa in birds is covered by two types of a keratinized stratified epithelium, namely the orthokeratinized and parakeratinized epithelia (Iwasaki et al. 1997; Jackowiak and Godynicki 2005; Jackowiak et al. 2011; Skieresz-Szewczyk and Jackowiak 2016). Microscopic and ultrastructural characteristics of these epithelia have been investigated in the bean goose, the domestic goose, and the domestic duck (Iwasaki et al. 1997; Skieresz-Szewczyk et al. 2014).

The orthokeratinized epithelium is present on the ventral surface of the lingual apex, where its cornified layer forms the plate of the lingual nail and also covers the mechanical papillae (Homberger and Brush 1986; Homberger and Meyers 1989; McLelland 1990; Iwasaki et al. 1997; Jackowiak and Godynicki 2005; Jackowiak et al. 2011; Skieresz-Szewczyk and Jackowiak 2016). Both the lingual nail and mechanical papillae are involved in food intake by pecking and grazing, determine the food transport to the esophagus, and prevent loss of food from the oral cavity.

The parakeratinized epithelium covers the dorsal surface of the tongue, responsible for the passive transport of food to the esophagus (Iwasaki et al. 1997; Jackowiak and Godynicki 2005; Jackowiak et al. 2011; Skieresz-Szewczyk and Jackowiak 2016). Birds from the order Anseriformes are characterized by two methods of food transport to the esophagus, i.e., the over-tongue and under-tongue transport (Kooloos 1986; Zweers et al. 1997; Van der Leeuw et al. 2003). Over-tongue transport is characteristic for goose (Kooloos 1986; Zweers et al. 1997; Van der Leeuw et al. 2003). Food in goose is moved along the median groove over the surface of the apex, body, and the lingual prominence, and these parts of the tongue are covered with parakeratinized epithelium (Iwasaki et al. 1997; Jackowiak et al. 2011). In the duck the manner is different, consisting in the so-called under-tongue transport (Kooloos 1986; Zweers et al. 1997; Van der Leeuw et al. 2003). Upon uptake, food is moved over the surface of the apex and the body of the tongue along the median groove, while further on, at the mid-length of the lingual body, i.e., in the area of the lingual comb, it is separated laterally into two parts and transported under the elevated margins of the anterio-lateral part of the lingual prominence of the tongue and next along the lateral surfaces of the lingual prominence. Due to the mechanism of food transport differing from that in the domestic goose, in the domestic duck, only the dorsal surface of the apex and the body of the tongue are covered by the stratified parakeratinized epithelium (Skieresz-Szewczyk and Jackowiak 2016). The presence of the parakeratinized epithelium over large areas of the dorsal surface of the tongue in birds is a natural and widespread phenomenon.

The aim of the study is to describe for the first time the sequence of micro- and ultrastructural changes in the epithelium on the dorsal surface of the lingual body in the domestic duck between days 9 and 25 of incubation. The results will be used to define developmental stages in the parakeratinized epithelium, identify the onset of formation of its keratinized layer, and assess the readiness of the epithelium to serve protective functions during food transport to the esophagus. At the same time, the recorded observations will be used to analyze the development of two types of keratinized epithelia, i.e., the para- and orthokeratinized epithelium, in the oral cavity in birds.

## Materials and methods

The present study according to Polish law and the EU directive no 2010/63/EU do not require approval of the Local Ethical Committee for Experiments on Animals in Poznan.

Incubation of the 78 fertilized eggs of the domestic duck and procedure of the removing the embryos from eggs were proceed according to protocol by Skieresz-Szewczyk et al. (2012).

Four tongues collected at 24-h intervals between the 9th and 25th days of incubation were dissected and subsequently fixed in 10% formalin. After 48 h of fixation, two tongues were prepared for light microscopic observations (LM) and two other tongues were prepared for examination under a scanning electron microscope (SEM).

Tissue samples for LM study were dehydrated in a series of increasing ethanol concentrations (70–96%) and routinely embedded in Paraplast® (Sigma-Aldrich, Germany). Paraplast blocks were cut into sections of 4.5–5 μm. Histological sections were stained using the Masson–Goldner trichrome staining technique (Romeis 1989). The sections were examined using an Axioscope2plus light microscope (Zeiss, Germany).

Histological measurements of the height of the epithelium, height of the superficial layer, and keratinized layer proceeded according to protocol of Skieresz-Szewczyk et al. (2018) by using Multiscan computer morphometric system (ver. 10.2, CSS, Warsaw, Poland). Statistical analyses of each morphological feature covered the following parameters: the mean value (*X*) with standard deviation (SD), the minimum value (min), and the maximum value (max). The *t* test for independent samples (level of significance *α* = 0.05) was used to determine the statistical significance of differences in mean values of the height of the epithelium between the particular days of incubation. The statistical analyses proceeded using the Statistica software (ver. 12.5, StatSoft, Poland).

Tissue samples for SEM study were dehydrated in a series of increasing ethanol concentrations (70–99.8%) for 15 min, then in a mixture of 96% ethanol and acetone (proportion 1:1) for 10 min and in acetone for 5 min. The tissue samples were then dried at critical point using CO_2_ (Critical Point Dryer K850, EMITECH). All samples were mounted on aluminum stubs covered with carbon tabs, sputtered with gold with 10 μm (Sputter Coater S 150B, EDWARDS) and observed under the SEM LEO 435 VP microscope (ZEISS) at an accelerating voltage of 10–15 kV.

Two dissected tongues from 12th, 19th, 20th, 22nd, and 24th days of incubation were fixed in a 2.5% solution of glutaraldehyde (Serva Electrophoresis, Germany) in cacodylate buffer (pH = 7.2) (Sigma-Aldrich, Germany) and then were fixed in a 4% osmium tetroxide (Sigma-Aldrich, Germany) solution in cacodylate buffer (pH = 7.2) at a temperature of 0–4 °C. Fixed tissue samples were dehydrated in a series of increasing ethanol concentrations (30–96%), a mixture of ethanol and propylene oxide (proportion 1:1), and pure propylene oxide at a temperature of 0–4 °C. Samples were embedded in Epon 812 epoxy resin (Serva Electrophoresis, Germany) with 2% DMP-30 (Serva Electrophoresis, Germany). Polymerization of the resin was run for 3 days at a temperature of 37 °C, 45 °C, and 60 °C. The sections of 70 nm were counter stained with 2% aqueous uranyl acetate and lead citrate. Observations and the photographic documentation were taken under a JEM1200 EX II transmission electron microscope (JEOL Co.).

Hamburger–Hamilton (HH) developmental stages (Hamburger and Hamilton 1951) are assigned to the particular days of incubation according to observation made by Skieresz-Szewczyk et al. (2018).

## Results

### Days 9 to 14 of incubation (stage HH 32/33 to 39)

**Fig. 1:**
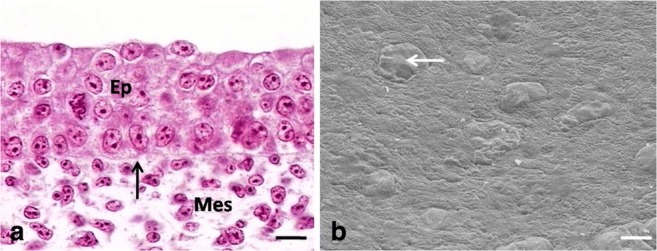
**a** 13th day of incubation. Cross-section through the epithelium (Ep) of the lingual body in the domestic duck. The arrow points towards the basal membrane. Mes, mesenchyme. LM, scale bar = 10 μm. **b** 12th day of incubation. Higher magnification of the flat surface of epithelial cells. The arrow points towards the slightly convex superficial cell. SEM, scale bar = 10 μm

The epithelium on the dorsal surface of the lingual body was composed of 6–7 layers of undifferentiated cells (Fig. 1a). The mean height of the epithelium on the dorsal surface of the lingual body at day 12 of incubation was 33.0 μm (Table 1). There was a statistically significant difference in the height of the epithelium between days 9, 10, 11, and 12. In contrast, no significant differences were found in the height of the epithelium between days 12, 13, and 14 of incubation.

**Table 1:** Height of the epithelium on the dorsal surface of the lingual body and its superficial layer and keratinized layer during embryonic development in the domestic duck

Day of incubation	Height of the epithelium (μm) *X* min.–max. SD	Height of the superficial layer (μm) *X* min.–max. SD	Height of the keratinized layer (μm) *X* min.–max. SD
9	38.0 29.7–44.7 3.7	–	–
10	35.5 27.9–43.2 3.4	–	–
11	30.9 23.1–39.4 4.4	–	–
12	33.0 21.2- 47.3 6.6	–	–
13	30.1 20.0–41.2 5.8	–	–
14	30.0 20.3–42.6 5.5	–	–
15	29.2 20.0- 42.6 6.7	–	–
16	31.4 23.8- 49.4 6.4	–	–
17	34.1 21.5–49.4 7.8	–	–
18	34.2 22.6–45.6 6.2	–	–
19	40.3 30.6- 49.1 5.5	–	–
20	40.2 23.2–55.9 8.8	3.5 2.4–5.6 0.8	–
21	49.0 35.3–78.8 13.1	3.8 3.3–4.1 0.4	–
22	77.2 53.2–98.8 12.3	6.3 5.0–7.4 0.8	–
23	85.0 55.3–109.6 13.2	–	3.3 2.7–4.4 0.7
24	121.8 91.0 -171.2 23.8	–	7.5 6.2–8.6 0.9
25	149.1 98.3–196.6 26.1	–	9.8 8.6–11.2 0.9

**Fig. 2:**
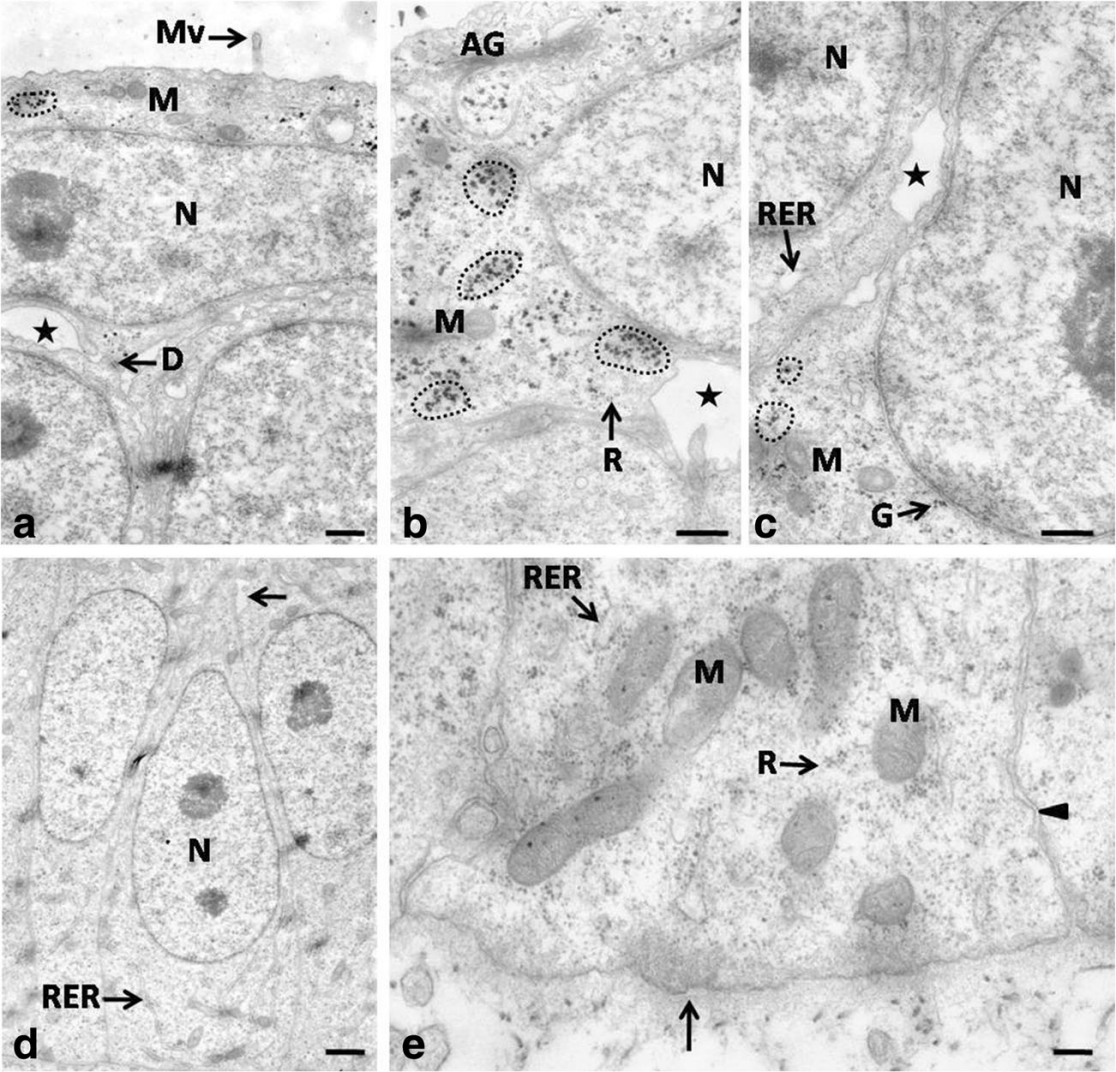
**a** 12th day of incubation. Transmission electron micrograph of the superficial cell. The asterisk points the intercellular space. The dotted line indicates the aggregates of glycogen. N, nucleus; Mv, microvilli; D, desmosome; M, mitochondrion. TEM, scale bar = 500 nm. **b** 12th day of incubation. Transmission electron micrograph of the superficial cell. The asterisk points the intercellular space. The dotted line indicates the aggregates of glycogen. AG, Golgi apparatus; N, nucleus; M, mitochondrion; R, single ribosomes. TEM, scale bar = 500 nm. **c** 12th day of incubation. Transmission electron micrograph of the intermediate cell. The asterisk points the intercellular space. The dotted line indicates the aggregates of glycogen. G, single granules of glycogen; N, nucleus; M, mitochondrion; RER, rough endoplasmic reticulum. TEM, scale bar = 500 nm. **d** 12th day of incubation. Transmission electron micrograph of the basal cells. The arrow points the intercellular space. N, nucleus; RER, rough endoplasmic reticulum. TEM, scale bar = 1 μm. **e** 12th day of incubation. Transmission electron micrograph of the basal cells. The arrow points the continuous basal membrane. The arrowhead indicates cell membrane closely adjacent to each other. M, mitochondrion; R, single ribosomes; RER, rough endoplasmic reticulum. TEM, scale bar = 200 nm

The epithelium was laid on the electron-lucent basal membrane (Fig. 2e). Cells and their nuclei throughout the epithelium were oval in shape and only some cells and nuclei were round in outline (Fig. 1a). Cell nuclei had one to two nucleoli. The cell cytoplasm was dyed pale pink (Fig. 1a).

Cell nuclei in the basal part of the epithelium were arranged perpendicular to the basal membrane (Fig. 2d). Cell membranes, immediately above the basal membrane, were adjacent to one another, while slightly above them intercellular spaces of a mean width of 293.8 nm were present (Fig. 2d, e). In the cell cytoplasm underneath the cell nuclei, the mitochondria—oval or round in cross-section and arranged in groups—were observed, as well as singly scattered RER cisternae of a mean width of 78.9 nm and single free ribosomes (Fig. 2d, e).

Cell membranes in the middle part of the epithelium were adjacent to one another and only locally the intercellular spaces of a mean width of 340.0 nm were found (Fig. 2c). The cell cytoplasm contained mitochondria round in cross-section and forming a group, single RER cisternae of a mean width of 74.0 nm, as well as numerous single ribosomes (Fig. 2c). Single glycogen granules or glycogen aggregates round in outline with a mean diameter of 43.9 nm were also present in the cell cytoplasm (Fig. 2c).

Superficial cells had horizontally located, oval cell nuclei, which almost completely filled cell cytoplasm (Fig. 2a). Numerous intercellular spaces of a mean width of 628.7 nm and single desmosomes on the edges of the intercellular spaces were found between the cell membranes of neighboring cells (Fig. 2a, b). Cell cytoplasm contained single mitochondria, oval or round in cross-section, as well as the Golgi apparatus and single RER cisternae of a mean width of 88.2 nm (Fig. 2a, b). Aggregates of glycogen with a mean diameter ranging between 96.9 and 201.1 nm were arranged around the cell nucleus (Fig. 2a, b). Single, short microvilli were present on the cell surface (Fig. 2a). Observations under the scanning electron microscope revealed the flat surface of the superficial cells, which were round in outline, while only single cell was slightly convex (Fig. 1b).

### Days 15 to 19 of incubation (stage HH 39/40 to 41/42)

**Fig. 3:**
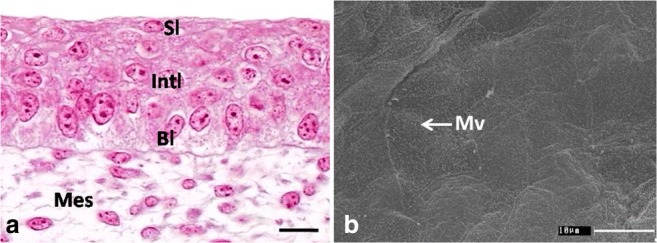
**a** 15th day of incubation. Cross-section through the epithelium (Ep) of the lingual body in the domestic duck divided into three layers: basal layer (Bl), intermediate layer (Int), and superficial layer (Sl). Mes, mesenchyme. LM, scale bar = 10 μm. **b** 14th day of incubation. Higher magnification of the polygonal in shape superficial epithelial cells. Mv, microvilli. SEM

The epithelium on the dorsal surface of the lingual body was still composed of 6–7 cell layers, which were arranged in three layers: basal, intermediate, and superficial (Fig. 3a). At day 15, the mean height of the epithelium was 29.2 μm (Table 1). There was a statistically significant difference in the height of the epithelium between days 15 and 16 and between days 18 and 19. The height of the epithelium between days 14, 15, 16, and 17 and between days 17 and 18 did not differ significantly.

**Fig. 4:**
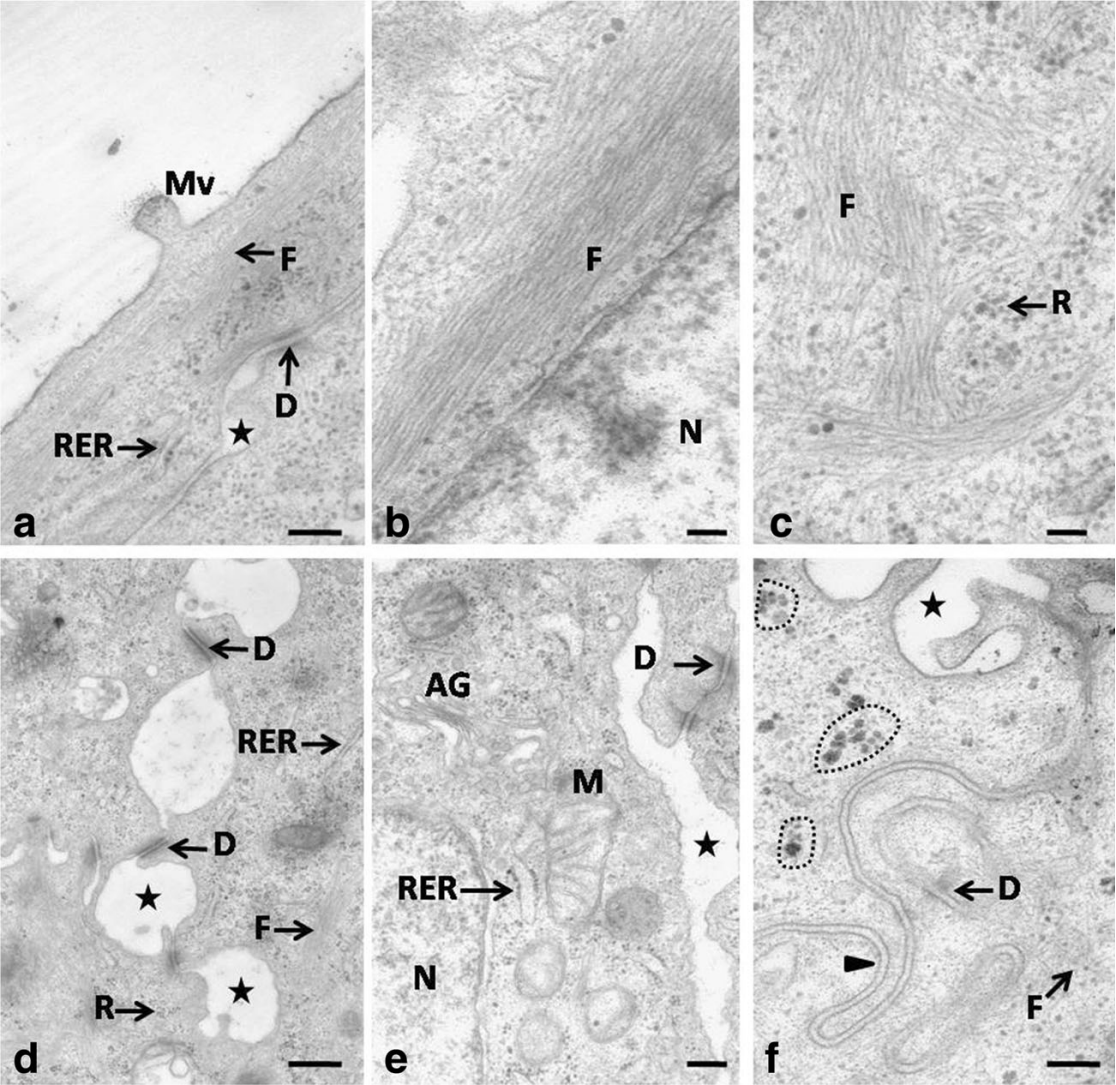
**a** 19th day of incubation. Transmission electron micrograph of the cell in the superficial layer. The asterisk points the intercellular space. D, desmosome; Mv, microvilli; F, filaments of cytoskeleton; RER, rough endoplasmic reticulum. TEM, scale bar = 200 nm. **b** 19th day of incubation. Transmission electron micrograph of the cell cytoplasm in the superficial layer. F, filaments of cytoskeleton; N, nucleus. TEM, scale bar = 100 nm. **c** 19th day of incubation. Transmission electron micrograph of the cell cytoplasm in the superficial layer. F, filaments of cytoskeleton; R, single ribosomes. TEM, scale bar = 100 nm. **d** 19th day of incubation. Transmission electron micrograph of the cell in the intermediate layer. Asterisks point wide intercellular spaces. D, desmosome; F, filaments of cytoskeleton; R, single ribosomes; RER, rough endoplasmic reticulum. TEM, scale bar = 500 nm. **e** 19th day of incubation. Transmission electron micrograph of the cell cytoplasm in the intermediate layer. The asterisk points the intercellular spaces. AG, Golgi apparatus; M, mitochondrion; R, single ribosomes; RER, rough endoplasmic reticulum. TEM, scale bar = 200 nm. **f** 19th day of incubation. Transmission electron micrograph of the cell cytoplasm in the basal layer. The asterisk points the intercellular spaces. The arrowhead points the adherent cell membranes forming cell membrane invagination. The dotted line indicates the aggregates of glycogen. D, desmosome; F, filaments of cytoskeleton. TEM, scale bar = 200 nm

The shape of cells in the basal layer was similar to that in the previous investigated embryonic period (Fig. 3a). The whole cell cytoplasm contained single, thin filaments of the cytoskeleton arranged in various directions and numerous single ribosomes (Fig. 4f). Glycogen grains formed aggregates of a mean diameter of 49.9 nm (Fig. 4f). Cell membranes of neighboring cells closely adhered to one another, forming mutual invaginations with single desmosomes (Fig. 4f). The intercellular spaces with an average width of 247.5 nm were observed occasionally (Fig. 4f).

The intermediate epithelial layer was composed of polygonal cells with oval or round cell nuclei arranged horizontally (Fig. 3a). The cell nucleus had one to two nucleoli. The whole cell cytoplasm contained oval or round, single mitochondria, RER cisternae of a mean width of 46.6 nm, a single Golgi apparatus, numerous free ribosomes, and single, thin filaments of the cytoskeleton interlaced in various directions (Fig. 4d, e). Numerous and wide intercellular spaces had a mean width of 865.3 nm (Fig. 4d, e). Cell membranes between intercellular spaces adhered closely to one another and were connected with desmosomes (Fig. 4d, e). On average, 5–10 desmosomes were observed in the cell cross-section.

The superficial layer was composed of two layers of flat cells with oval, horizontally arranged cell nuclei (Fig. 3a). SEM observations of the epithelial surface showed that cells were polygonal in shape and slightly elongated (Fig. 3b). Filaments of the cytoskeleton were arranged along the cell nuclei or interlaced in various directions in the cell cytoplasm (Fig. 4a–c). Numerous free ribosomes and single RER cisternae of a mean width of 43.0 nm were observed between filaments of the cytoskeleton (Fig. 4a–c). On the cell surface single, short microvilli were present (Figs. 3b and 4a). Cell membranes of neighboring cells closely adhered to one another and were connected with the desmosome (Fig. 4a). On average, 5–10 desmosomes were observed on the cell circumference. Single, narrow intercellular spaces had an average width of 92.2 nm (Fig. 4a).

### Days 20 to 22 of incubation (stage HH 41/42 to 43/44)

**Fig. 5:**
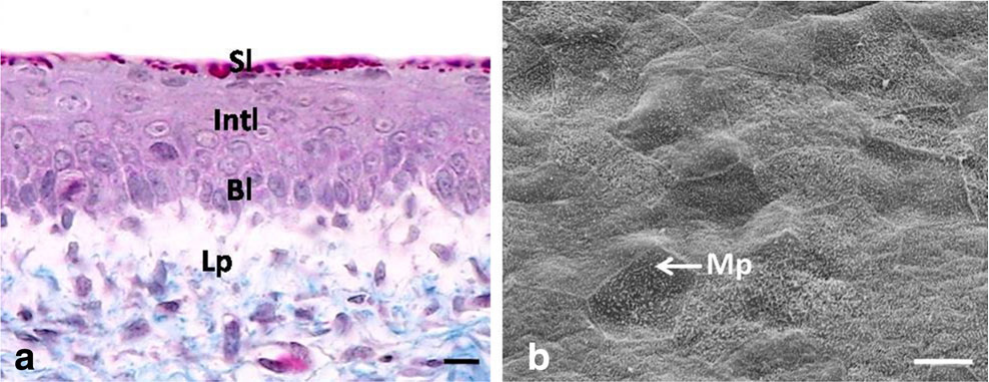
**a** 20th day of incubation. Cross-section through the epithelium (Ep) of the lingual body in the domestic duck divided into three layers: basal layer (Bl), intermediate layer (Int), and superficial layer (Sl) with red granules. Lp, lamina propria. LM, scale bar = 10 μm. **b** 20th day of incubation. Higher magnification of the polygonal in shape superficial epithelial cells with microprojection (Mp). SEM, scale bar = 100 μm

At the turn of days 20 and 21, the epithelium on the dorsal surface of the lingual body was composed of 8–9 cell layers (Fig. 5a). At day 20 of incubation, the average height of the epithelium was 40.2 μm (Table 1). The height of the epithelium between days 20 ad 21 differed significantly.

**Fig. 6:**
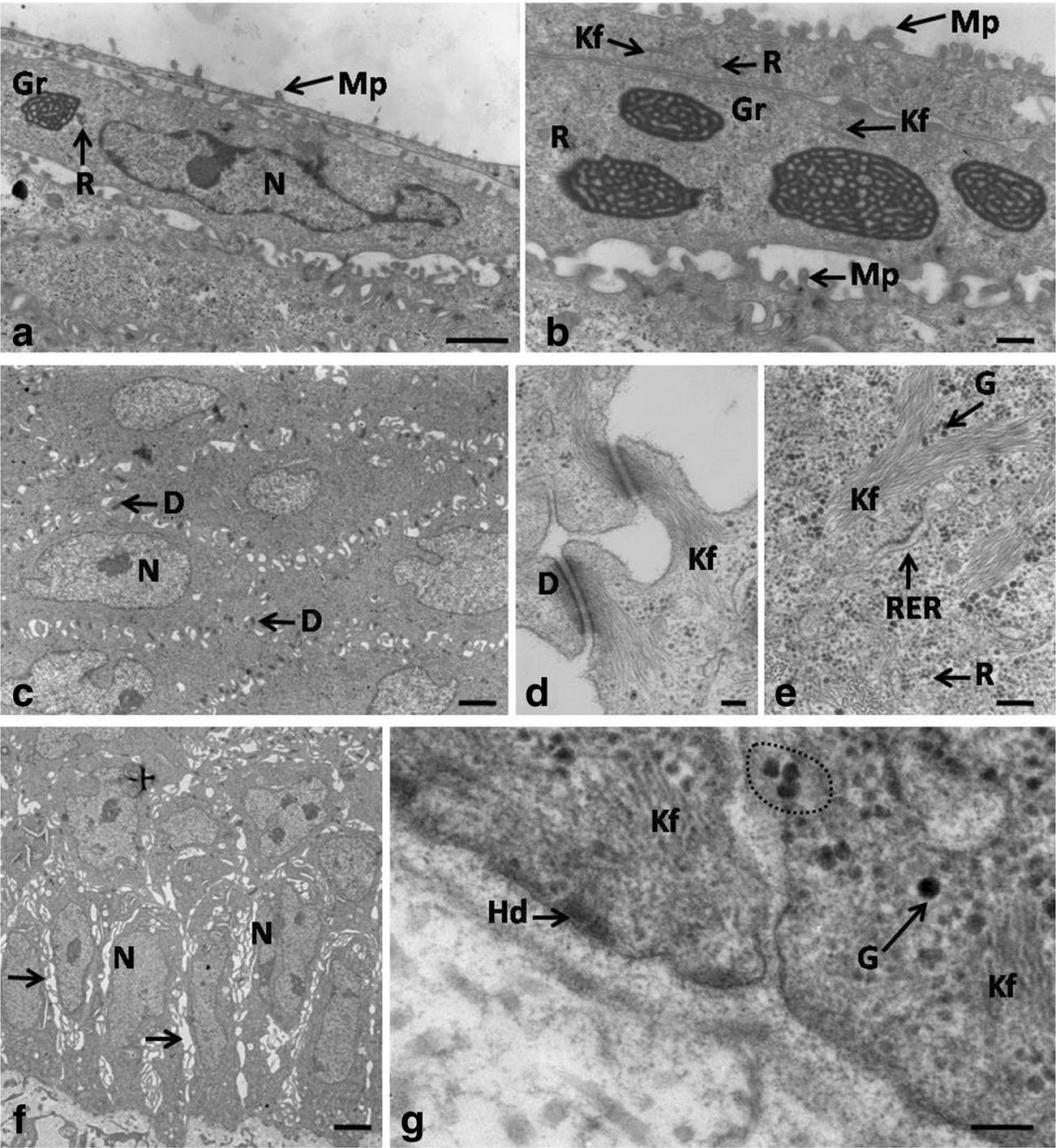
**a** 20th day of incubation. Transmission electron micrograph of the cells in the superficial layer. Gr, osmophilic granules; Mp, microprojection; N, nucleous; R, clusters of free ribosomes. TEM, scale bar = 1 μm. **b** 20th day of incubation. Transmission electron micrograph of the cells in the superficial layer. Gr, osmophilic granules; Mp, microprojection; N, nucleous; R, clusters of free ribosomes. TEM, scale bar = 500 nm. **c** 20th day of incubation. Transmission electron micrograph of the cells in the intermediate layer. N, nucleous; D, desmosome. TEM, scale bar = 2 μm. **d** 20th day of incubation. Transmission electron micrograph of the microprojection connected by desmosomes. Kf, keratin fibers. TEM, scale bar = 100 nm. **e** 20th day of incubation. Transmission electron micrograph of the cell cytoplasm in the intermediate layer. G, single grains of glycogen; Kf, keratin fibers; R, single ribosomes; RER, rough endoplasmic reticulum. TEM, scale bar = 200 nm. **f** 20th day of incubation. Transmission electron micrograph of the cells in the basal layer. Arrows point the intercellular spaces. N, nucleus. TEM, scale bar = 2 μm. **g** 20th day of incubation. Transmission electron micrograph of the cells in the basal layer. The dotted line indicates aggregates of glycogen. Hd, hemidesmosomes; Kf, keratin fibers. TEM, scale bar = 100 nm

Cells and cell nuclei in the basal layer were elliptical in shape and were connected with the basal membrane by hemidesmosomes (Fig. 6f, g). Intercellular spaces with a mean width of 495.7 nm were found between neighboring cells (Fig. 6f). The cell cytoplasm around the cell nucleus was filled with thin bundles of keratin fibers, between which numerous free ribosomes and single RER cisternae of 68.4 nm in mean width were found (Fig. 6g). Single grains of glycogen and aggregates of glycogen with a mean diameter of 59.5 nm were observed in the cell cytoplasm of the basal layer (Fig. 6g).

Most of the polygonally shaped cells in the intermediate layer had oval cell nuclei, wherein in single cells, the nucleus membrane formed invagination to karyoplasm (Fig. 6c). The whole cell cytoplasm was filled with electron dark keratin fibers arranged in various directions (Fig. 6e). Single RER cisternae with a mean width of 53.6 nm, clusters of the free ribosomes, and grains of glycogen were present between keratin fibers (Fig. 6e). The cell membrane of the polygonal cell in the intermediate layer formed microprojections connected by desmosomes (Fig. 6d). On the cross-section of a single cell, on average, 16–20 desmosomes were visible. Between neighboring cells, single intercellular spaces with a mean width of 384.4 nm were present (Fig. 6c).

The superficial layer was composed of 2–3 cell layers. Masson–Goldner trichrome staining revealed the presence of round and red grains in the cell cytoplasm of the two-cell layers immediately above the intermediate layer (Fig. 5a). The ultrastructural observations showed oval or round granules resembling a mesh-like structure composed of coarse filaments of osmophilic material with a mean width of 36.5 nm and electron-lucent material (Fig. 6a, b). The average diameter of the granules ranged between 221.6 and 827.7 nm. Cells with granules were devoid of cell nuclei or their cell nuclei were irregular in shape (Fig. 6a, b). The cytoplasm contained keratin fibers and clusters of the free ribosomes located around the osmophilic granules (Fig. 6a, b). Cells with osmophilic granules were covered with two flat cell layers that had no cell nuclei and their cell cytoplasm was filled with keratin fibers and clusters of the free ribosomes (Fig. 6a, b). The most superficial two-cell layers were clearly separated from lower lying cells and were undergoing exfoliation (Fig. 6a, b). Numerous and short microprojections were visible on the cell surface (Figs. 5b and 6a, b). The mean height of the superficial layer at day 20 of incubation was 3.5 μm (Table 1).

**Fig. 7:**
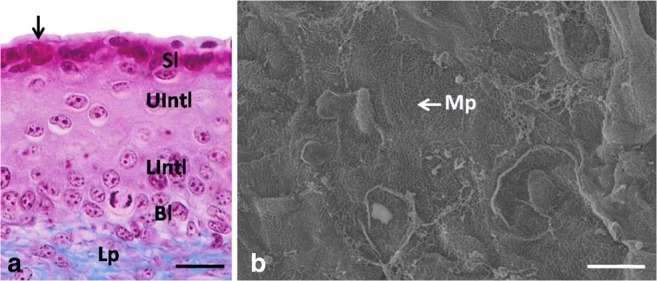
**a** 22nd day of incubation. Cross-section through the epithelium (Ep) of the lingual body in the domestic duck. The arrow points the red-colored granules. Bl, basal layer; Int, intermediate layer; Lp, lamina propria; LInt, lower part of the intermediate layer; Sl, superficial layer; UInt, upper part of the intermediate layer. LM, scale bar = 20 μm. **b** 22nd day of incubation. Higher magnification of the polygonal in shape superficial epithelial cells with microprojection (Mp). SEM, scale bar = 10 μm

At day 22 of incubation, the epithelium of the lingual body was composed of 10–11 cell layers (Fig. 7a). The mean height of the epithelium was 77.2 μm (Table 1). There is a statistically significant difference in the height of the epithelium between days 21 and 22 of incubation.

**Fig. 8:**
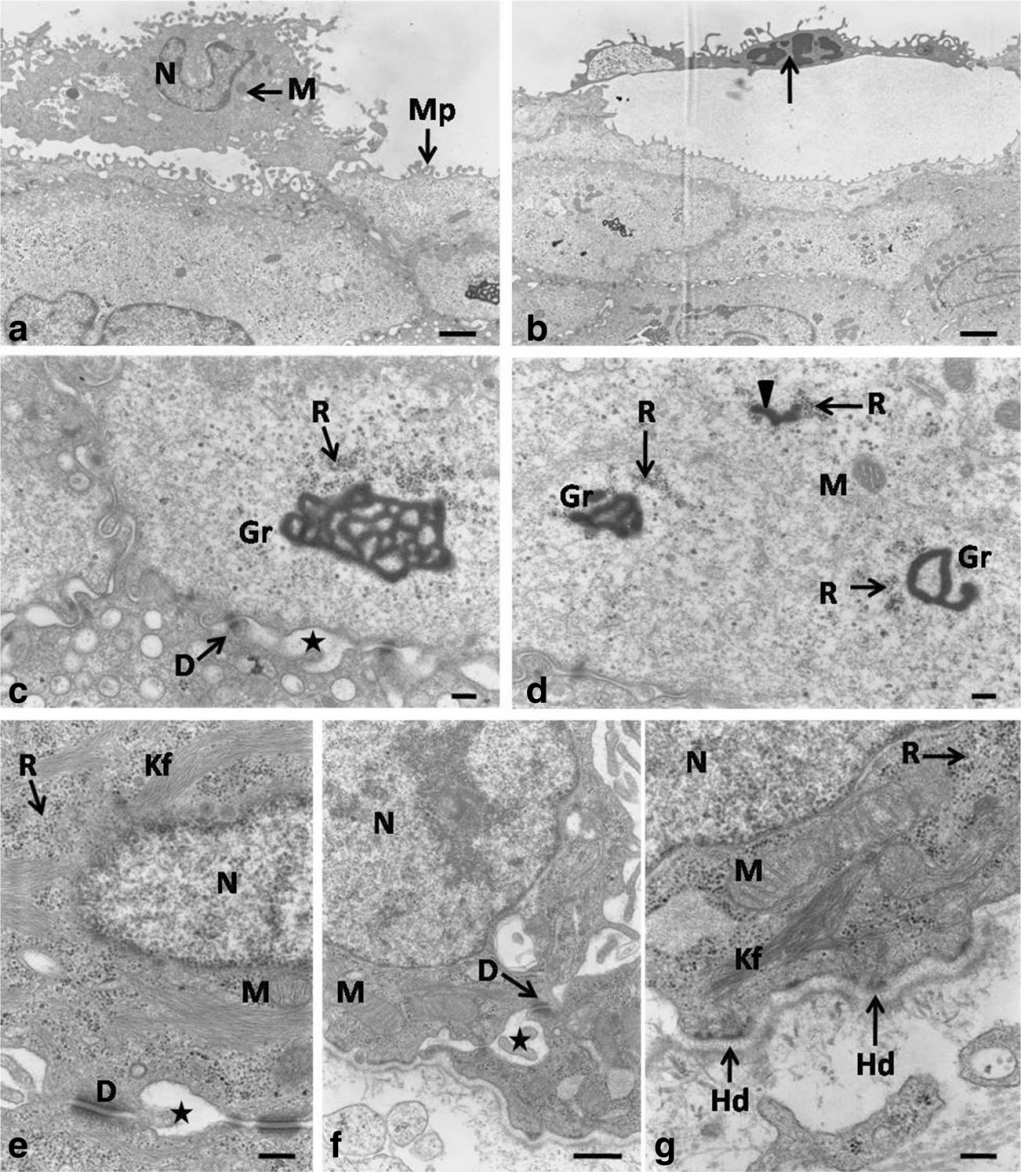
**a** 22th day of incubation. Transmission electron micrograph of the superficial layer with detached superficial cell. N, nucleous with invagination into karyoplasm; M, mitochondrion; Mp, microprojection. TEM, scale bar = 1 μm. **b** 22th day of incubation. Transmission electron micrograph of the cells in the superficial layer. The arrow points towards condensed nucleus. TEM, scale bar = 2 μm. **c** 22th day of incubation. Transmission electron micrograph of the cell cytoplasm in the superficial layer. The asterisk points the intercellular space. D, desmosome; Gr, osmophilic granules; R, clusters of free ribosomes. TEM, scale bar = 200 nm. **d** 22th day of incubation. Transmission electron micrograph of the cell cytoplasm in the superficial layer. The arrowhead points electron dark material. Gr, osmophilic granule; M, mitochondrion; R, clusters of free ribosomes. TEM, scale = 200 nm. **e** 22th day of incubation. Transmission electron micrograph of the cell cytoplasm in the lower part of the intermediate layer. The asterisk points the intercellular space. D, desmosome; M, mitochondrion; N, nucleous; Kf, keratin fibers; R, free ribosomes. TEM, scale bar = 200 nm. **f** 22th day of incubation. Transmission electron micrograph of the cells in the basal layer. The asterisk points the intercellular space. D, desmosome; M, mitochondrion; N, nucleous. TEM, scale bar = 500 nm. **g** 22th day of incubation. Transmission electron micrograph of the cells in the basal layer. Hd, hemidesmosomes; M, mitochondrion; N, nucleous; Kf, keratin fibers; R, free ribosomes. TEM, scale bar = 200 nm

Cells and their nuclei in the basal layer were oval in shape (Fig. 7a). Electron-dense keratin fibers and numerous free ribosomes filled the cell cytoplasm (Fig. 8g). The mitochondria—oval or round in cross-section—were found in the cell cytoplasm immediately above the basal membrane (Fig. 8f, g). Numerous intercellular spaces of 230.8 nm in mean width were observed (Fig. 8f). Desmosomes, in which the average number ranged between 16 and 20, were visible on the cell circumference (Fig. 8f).

LM observations allowed to distinguish, depending on the types of polygonal cells, two parts in the intermediate layer: the lower and upper parts (Fig. 8e). Cells in the lower part of the intermediate layer had oval, horizontally arranged cell nuclei and their cytoplasm after Masson–Goldner trichrome staining was dyed pink. Cells in the upper part of the intermediate layer had slightly flat cell nuclei and their cytoplasm, around the nucleus, was pale pink. TEM observation did not reveal significant differences in the ultrastructure between those two parts. Generally, electron-dense keratin fibers were arranged parallel to the nucleus membrane and between them free ribosomes, single RER cisternae, and single mitochondria, round or oval in the cross-section, were present (Fig. 8e). In the cross-section of a single cell, on average, 15–20 desmosomes were visible. Single intercellular spaces had a mean width of 179.7 nm (Fig. 8e).

The superficial layer was composed of 3–4 cell layers, in which the cytoplasm, after Masson–Goldner staining, was strongly dyed dark pink (Fig. 7a). Red granules were observed in single cells (Fig. 7a). Ultrastructural observations revealed the presence of osmophilic granules, in which the diameter ranged between 185.3 nm and 506.3 nm (Fig. 8c, d). Bands of the osmophilic material, surrounded by numerous free ribosomes and single mitochondria, were observed in the single cells (Fig. 8d). Some of the superficial cells had irregularly shaped cell nuclei, while single mitochondria, free ribosomes, and densely arranged electron light keratin fibers were present in the cell cytoplasm (Fig. 8a). Some of the superficial cells had nuclei with strongly condensed chromatin and the cell cytoplasm was devoid of cell organelles and filled with electron-dense keratin fibers (Fig. 8b). Cell membranes of the neighboring cells formed numerous invaginations and were connected by desmosomes (Fig. 8c). On average, 25–30 desmosomes were visible on the cell circumference. Single intercellular spaces had the mean width of 122.9 nm (Fig. 8c). Numerous microprojections were visible on the cell surface (Figs. 7b and 8a). The superficial cells peeled off as single cells (Fig. 7b). The average height of the superficial layer at 22 days was 6.3 μm (Table 1).

### Days 23 to 25 of incubation (stage HH 45 to 46)

**Fig. 9:**
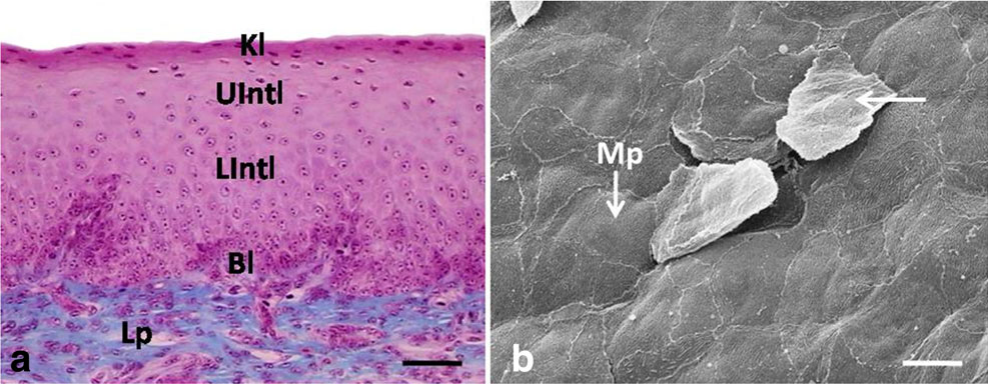
**a** 24th day incubation. Cross-section through the epithelium (Ep) of the lingual body in the domestic duck. Bl, basal layer; LInt, lower part of the intermediate layer; UInt, upper part of the intermediate layer; Lp, lamina propria; Kl, keratinized superficial layer. LM, scale bar = 35 μm. **b** 24th day incubation. Higher magnification of the polygonal in shape superficial epithelial cells with parallel arranged microprojection (Mp). The white arrow points the desquamate superficial cell. SEM, scale bar = 10 μm

Between days 23 and 25 of incubation, the stratified multilayered epithelium of the lingual body was composed of 24–25 cell layers arranged in three layers: basal, intermediate, and keratinized (Fig. 9a). During the examined period, the superficial layer with red granules was atrophied. The average height of the epithelium increased to 149.1 μm at day 25 of incubation (Table 1). The height of the epithelium between days 23 and 24 differed significantly.

The microstructure of the basal layer and lower part of the intermediate layer did not change compared to the earlier stages of embryonic development.

**Fig. 10:**
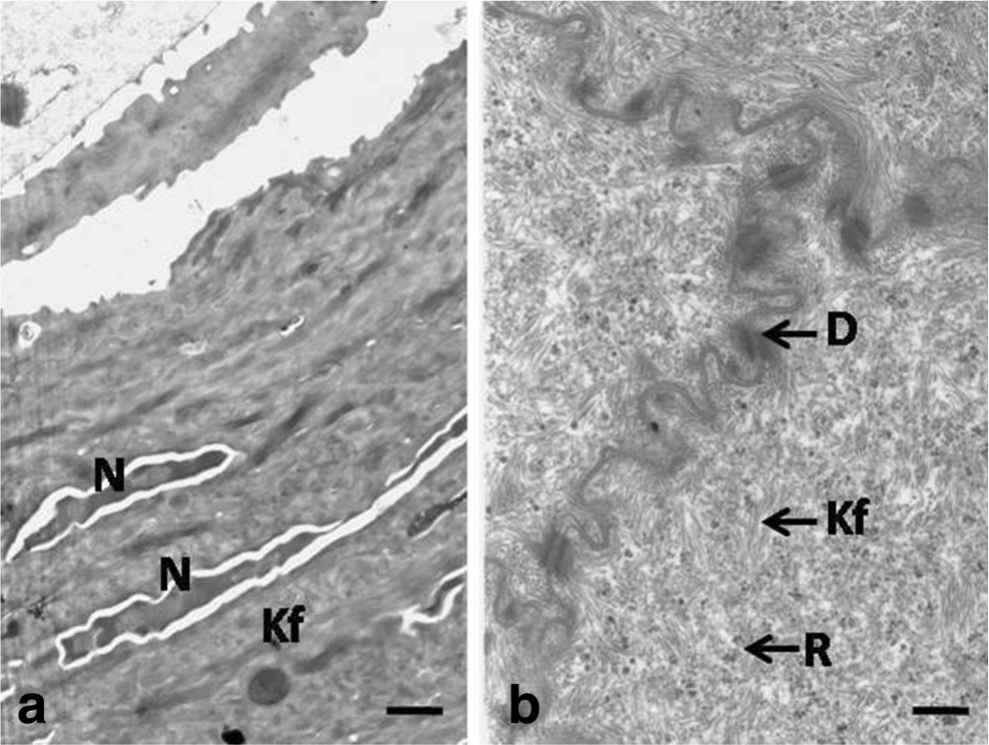
**a** 24th day incubation. Transmission electron micrograph of the keratinized layer. N, nucleous; Kf, keratin fibers. TEM, scale bar = 1 μm. **b** 24th day incubation. Transmission electron micrograph of the upper part of the intermediate layer. D, desmosome; Kf, keratin fibers; R, free ribosomes. TEM, scale bar = 200 nm

The polygonal cells of the upper part of the intermediate layer were flat and single cells were devoid of cell nuclei (Fig. 9a). The cell cytoplasm contained horizontally arranged electron-dense keratin fibers and ribosomes forming clusters of 3–4 ribosomes (Fig. 10b). Cell membranes of the neighboring cells formed numerous invaginations connected by desmosomes (Fig. 10b). Narrow intercellular spaces of 35.3 nm in mean width were present between neighboring cells. On average, 25–30 desmosomes were visible on the cross-section of a single cell.

The keratinized layer was composed of 3–4 cell layers, in which the cytoplasm after Masson–Goldner trichrome staining was dyed dark pink (Fig. 9a). Ultrastructural observations revealed that the cell cytoplasm was filled with electron-dense keratin fibers, arranged around the cell nucleus, as well as free ribosomes (Fig. 10a). On average, half of the keratinized cells were devoid of the cell nucleus and in the others the nucleus had condensed chromatin (Figs. 9a and 10a). Narrow intercellular spaces with a mean width of 40.7 nm were present between neighboring cells. Surface observations under the scanning electron microscope reveled that flat superficial cells exfoliate as single scales (Fig. 9b). Regularly arranged microprojections were visible on the surface of lower lying cells (Fig. 9b). The mean thickness of the keratinized layer at day 25 of incubation was 9.8 μm (Table 1).

## Discussion

Beside the orthokeratinized epithelium, the parakeratinized epithelium is one of the two types of keratinized epithelia covering the mucous membrane of the oral cavity in birds (Iwasaki et al. 1997; Jackowiak and Godynicki 2005; Jackowiak et al. 2011; Skieresz-Szewczyk and Jackowiak 2016). Previous microscopic and ultrastructural analyses of parakeratinized epithelium of the tongue in the domestic duck showed that the epithelium is composed of three layers, i.e., basal, intermediate, and keratinized (Skieresz-Szewczyk et al. 2014). A specific characteristic of the intermediate layer is connected with the presence of two diverse zones. In the lower zone, oval, horizontally arranged nuclei are found, whereas in the upper zone they are flat. A feature distinguishing the parakeratinized epithelium from the orthokeratinized epithelium is the presence of nuclei with condensed chromatin in the keratinized layer, observed in the former epithelium type.

Previous studies by Skieresz-Szewczyk and Jackowiak (2017) and Skieresz-Szewczyk et al. (2018) concerning the development of orthokeratinized epithelium identified three developmental stages of the epithelium, i.e., embryonic, transformation, and pre-hatching stages. The same developmental stages were observed in the current investigations on the development of parakeratinized epithelium of the body of the tongue in the domestic duck.

A comparison of the present results with earlier observations of development of the orthokeratinized epithelium, presented in Table 2, revealed essential differences in timing of the particular developmental stages and also specific traits in epithelial cells in the particular developmental stages.

**Table 2:** Comparative analyses of the development of the parakeratinized and orthokeratinized epithelium in the oral cavity in birds

Developmental stage	Parakeratinized epithelium	Orthokeratinized epithelium, ventral surface of the lingual apex (Skieresz-Szewczyk et al. 2018)	Orthokeratinized epithelium, small conical papillae (Skieresz-Szewczyk and Jackowiak 2017)	Orthokeratinized epithelium, large conical papillae (Skieresz-Szewczyk and Jackowiak 2017)	Orthokeratinized epithelium, conical papillae of the lingual prominence (Skieresz-Szewczyk and Jackowiak 2017)
Embryonic stage	9–14 days	9–13 days	16–19 days	11–13 days	16 days
	Undifferentiated, embryonic epithelial cells with: - single Golgi apparatus and cisternae of rER, numerous free ribosomes, and glycogen aggregates - numerous mitochondria arranged in clusters around cell nuclei or in the subnuclear region of the basal cells Occurrence of the microvilli on the surface of superficial cells				
Transformation stage	15–22 days	14–20 days	18–25 days	14–20 days	17–22 days
	Morphological differentiation of the embryonic cells and arrangement of cells into three layers: basal, intermediate, and superficial layer Presence of the microvilli on the surface of superficial cells				
	Presence of specific red granules in cell cytoplasm in the superficial layer		Lack of red granules	Lack of red granules	Presence of specific red granules in cell cytoplasm in the superficial layer
	Red granules have a mesh-like structure and are composed of osmophilic coarse filaments Accumulation of clusters of ribosomes around the mesh-like granules, which may participate in the formation of granules		–	–	No data
	Size of mesh-like granules decreases Number of layers of superficial cells with granules do not change	Size of periderm granules increases Number of layers of superficial cells with granules also increases	–	–	No data
Pre-hatching stage	23–25 days	21–25 days	–	21–25 days	18–25 days
	Lack of the red granules/mesh-like granules		–	–	Lack of the red granules/mesh-like granules
	The keratinized layer develops after the periderm with periderm granules has atrophied	The cornified layer is formed at the time when the epithelium is already covered by periderm with periderm granules	The cornified layer is not formed	The cornified layer is formed at the same time as the superficial layer	The cornified layer develops after the periderm with periderm granules has atrophied
	Presence of microprojections on the surface of the keratinized cells	Presence of microridges on the surface of the cornified cells	–	Presence of microridges on the surface of the cornified cells	–

A characteristic, important feature for the parakeratinized epithelium and also for orthokeratinized epithelium in the oral cavity in birds, during the transformation stage, is the formation of red granules in the cell cytoplasm of the superficial cells (Skieresz-Szewczyk and Jackowiak 2017; Skieresz-Szewczyk et al. 2018). The ultrastructural observations made by Skieresz-Szewczyk et al. (2018) on the orthokeratinized epithelium revealed that the red granules have a mesh-like structure composed of osmophilic coarse filaments. The present study confirms that observation. Previously, similar mesh-like structures were recorded in the periderm during the development of the interfollicular skin, scales, claws, and feathers in reptiles and birds and were named periderm granules (Alibardi 1998, 2002, 2009a, b, 2014; Alibardi et al. 2015). The diameter of the coarse filaments forming periderm granules varies between taxa. Periderm granules in the periderm of the interfollicular skin in birds are composed of coarse filaments of 20–40 nm in diameter, while in the periderm of the scales and beak the diameter of coarse filaments is 30–50 nm (Alibardi 2002; Alibardi et al. 2015). In comparison, the diameter of periderm granules in reptiles ranges between 20 and 25 nm (Alibardi 1998). In the orthokeratinized epithelium of the tongue, coarse filaments are 30–40 nm in diameter. This study showed that the diameter of the coarse filaments in the parakeratinized epithelium is similar to that in the orthokeratinized epithelium, approximately 35 nm. In mammals no periderm granules are formed in the periderm during embryonic development of the epidermis (Akiyama et al. 1999).

The stratified parakeratinized epithelium serves a protective function during food transport to the esophagus (Jackowiak et al. 2011; Skieresz-Szewczyk and Jackowiak 2016). Proper development of the parakeratinized epithelium of the tongue in the domestic duck is crucial for food uptake by young birds. At hatching in the domestic duck the filtration apparatus formed of small and large conical and filiform papillae is not fully developed and food intake by filtration is not possible (Skieresz-Szewczyk et al. 2014; Skieresz-Szewczyk and Jackowiak 2016). Thus, as stated by Van der Leeuw et al. (2003), immediately after hatching, young birds collect solid food or food immersed in water by pecking. For this reason, it is crucial to develop the parakeratinized epithelium of the body of the tongue, over which food is transported to the esophagus.

The protective barrier of stratified epithelia is formed by mechanical intercellular bonds, i.e., desmosomes, and the keratinized layer. This study showed that during the embryonic development, first of all during the transformation stage, but also in the pre-hatching stage, the impermeable protective epithelium barrier is gradually formed. This study showed that at the beginning of the transformation stage, on the edges of wide intercellular spaces in the basal and intermediate layers of the epithelium, single desmosomes are formed. From day 20 of incubation, i.e., towards the transformation stage, the width of intercellular spaces in the basal and intermediate layers of the epithelium was reduced. In the basal layer of the epithelium, the width of intercellular spaces decreased on average by half, while in the intermediate layer the reduction was over 4-fold. At the same time, on average, a 4-fold increase was found in the number of desmosomes observed at the cross-sections of cells in the basal and intermediate layers of the epithelium. Meanwhile, in the superficial layer, the number of desmosomes increased by as much as 6-fold. Additionally, during the transformation stage, the first protective layer of the epithelium in the form of periderm is formed. Similar observations on the decrease in the width of intercellular spaces, an increase in the number of desmosomes, and formation of periderm were recorded in the orthokeratinized epithelium (Skieresz-Szewczyk et al. 2018). In the pre-hatching stage in the intermediate layer of parakeratinized epithelium, a further reduction is observed in the width of intercellular spaces and an increase is recorded in the number of desmosomes along with the initiation of the formation of the proper protective barrier of stratified epithelium, i.e., the keratinized layer. Finally, from the transformation stage until the end of the pre-hatching stage, the width of intercellular spaces in the intermediate layer of the epithelium is reduced over 20-fold and the number of desmosomes increases on average 6-fold. Neighboring cells both in the intermediate and the newly formed keratinized layer are separated with narrow gaps, and cell membranes undergo mutual invaginations and are bound with desmosomes. In summary, the parakeratinized epithelium on the body of the tongue in the domestic duck immediately before hatching is structurally ready to serve the protective function during food transport to the esophagus.

Differentiation of the embryonic epithelium in the oral cavity in stratified epithelium is a process requiring expenditure of energy. According to McFall and Kraus (1965), glycogen is a storage material necessary for morphological changes. This study showed that aggregates of glycogen grains are found in the cytoplasm of epithelial cells both in the embryonic and transformation stages. In the case of orthokeratinized epithelium of the tongue, glycogen grains were observed only in the cytoplasm of cells in the embryonic stage (Skieresz-Szewczyk et al. 2018). In view of the length of the transformation stage of parakeratinized epithelium, until day 22 of incubation, and not until day 20 as in the case of orthokeratinized epithelium, the presence of glycogen grains at the transformation stage of parakeratinized epithelium may indicate that its development requires greater energy reserves to undergo structural changes than in the case with orthokeratinized epithelium.

Towards the end of the transformation stage at about day 20 and in the pre-hatching stage on the surface of cells in the superficial and keratinized layers, cytoplasmic microprojections, arranged regularly in relation to one another, were observed. To date, literature sources have termed similar structures microridges. Microridges are considered to be structures formed during the keratinization process, and they are characteristic features of desquamated mature keratinocytes (Iwasaki 1993; Iwasaki et al. 1999). Ultrastructural observations in this study showed that microprojections are found in sites corresponding to mutual invaginations of cell membranes of neighboring epithelial cells and locations of desmosomes. Another interpretation should be taken into account: that microprojections and/or microridges are sites of former intercellular connection, visible after cell exfoliation. Parallelly arranged microprojections and/or microridges present on the surface of the keratinized cells may provide better adhesion both to mucus produced by the mucous glands of the oral cavity and to bacteria (Sperry and Wassersug 1976).

To conclude, the parakeratinized epithelium at the end of incubation resembles the epithelium in adult birds. A comparative analysis of the development of both keratinized epithelia in the oral cavity, namely the parakeratinized and orthokeratinized epithelium in birds, showed that the periderm with specific osmophilic periderm granules is a common feature characteristic for these two types of keratinized epithelia in the oral cavity of birds but the formation of the keratinized/cornified layer is different and, according to Skieresz-Szewczyk and Jackowiak (2017) and Skieresz-Szewczyk et al. (2018), exhibits three different variants. The first one is that the cornified layer is formed at the same time as the formation of the superficial layer with periderm granules (Skieresz-Szewczyk and Jackowiak 2017). In the second one, the cornified layer develops when the epithelium is already covered by a superficial layer with periderm granules (Skieresz-Szewczyk et al. 2018). And the third one, observed in the studied parakeratinized epithelium, reveals that the cornified layer is formed immediately after exfoliation of the superficial layer.
